# Lost in a Sea of Information: The Microplastics Publication Deluge

**DOI:** 10.1021/acs.est.6c01796

**Published:** 2026-05-28

**Authors:** Nanna B. Hartmann, David Mennekes, Martin Wagner

**Affiliations:** † 5205 Technical University of Denmark, Department of Environmental and Resource Engineering (DTU Sustain), 2800 Kgs. Lyngby, Denmark; ‡ 27219 ETH Zürich, Department of Environmental Systems Science, 8092 Zürich, Switzerland; § Norwegian University of Science and Technology (NTNU), Department of Biology, Trondheim 7491, Norway

**Keywords:** Plastic pollution, Information overload, Overpublication, Evidence synthesis, Study quality, Incentive structures, Agenda setting

## Abstract

Environmental microplastics research has expanded rapidly over the past two decades. Although the presence of microplastics in the ocean were first described more than 50 years ago, consistent scientific, public, and regulatory attention has emerged only in the last 15–20 years. Since then, publication output has grown almost exponentially, reaching a point where the volume of literature exceeds the community’s capacity to effectively track, synthesize, and mobilize new information into actionable knowledge. In this Perspective, we describe what we term the *publication deluge*: a state in which continued accumulation of studies generates information overload and fog rather than clarity. We argue that this situation comes with a risk of blurring established understanding, reopening debate around facts with established consensus, and delaying progress toward prioritization and long-term solutions. Using a conceptual framework that distinguishes between problem stabilization, refinement, and solution-oriented understanding, we discuss structural drivers of this lock-in and outline concrete actions that authors, editors, publishers, institutions, and funders can take to push the field toward more synthetic, integrative, and actionable knowledge.

## Graphical abstract

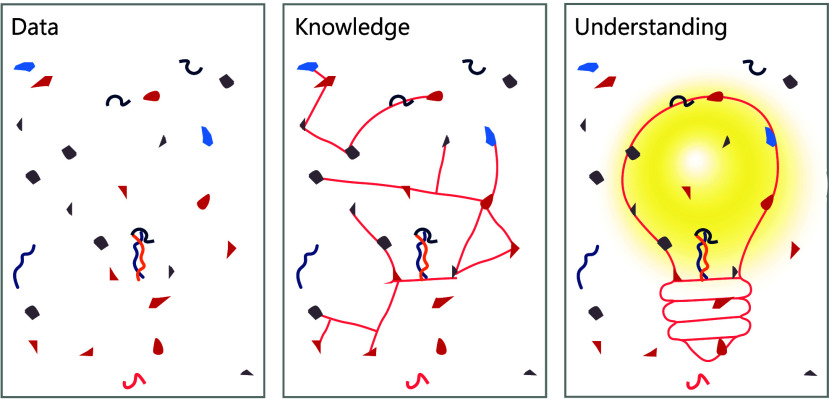

## 1 The Rise of the Publication Deluge

From early findings on the presence of microplastics in the oceans [refs 1–4 and others] and examples of biological uptake [ref 5 and others] research has branched out into a number of subfields, covering the occurrence of microplastics in all environmental compartments as well as their impacts on a multitude of species and ecosystems. Ecotoxicology is a main driver in microplastics research, but the field has also seen major advances in analytical methods to detect microplastics, approaches to model their fate as well as the addition of social, economic and political perspectives.

As a result of this development and fueled by public and political concerns and scientific curiosity, the literature on microplastics has expanded rapidly in recent years [Figure 1,^6,7^ ]. This phenomenon, observed across scientific disciplines, has been referred to by others as a publication “explosion” ^8,9^ or as a “flood” of scientific publications.^10^ Here, we adopt the term “publication deluge”.^10–12^ On one hand, such growth in the literature is beneficial as continued data generation is undoubtedly needed to better understand the problem. It also signifies the diversification of the field far beyond marine science and ecotoxicology as well as sustained support by funding agencies. Finally, it may be a result of more inclusive publication practices, which—if so—represents a clearly positive development, particularly in broadening participation beyond traditional Global North research communities.^13,14^ On the other hand, the exponential growth, or deluge, in the microplastics literature poses numerous challenges.^15^

**Figure 1:**
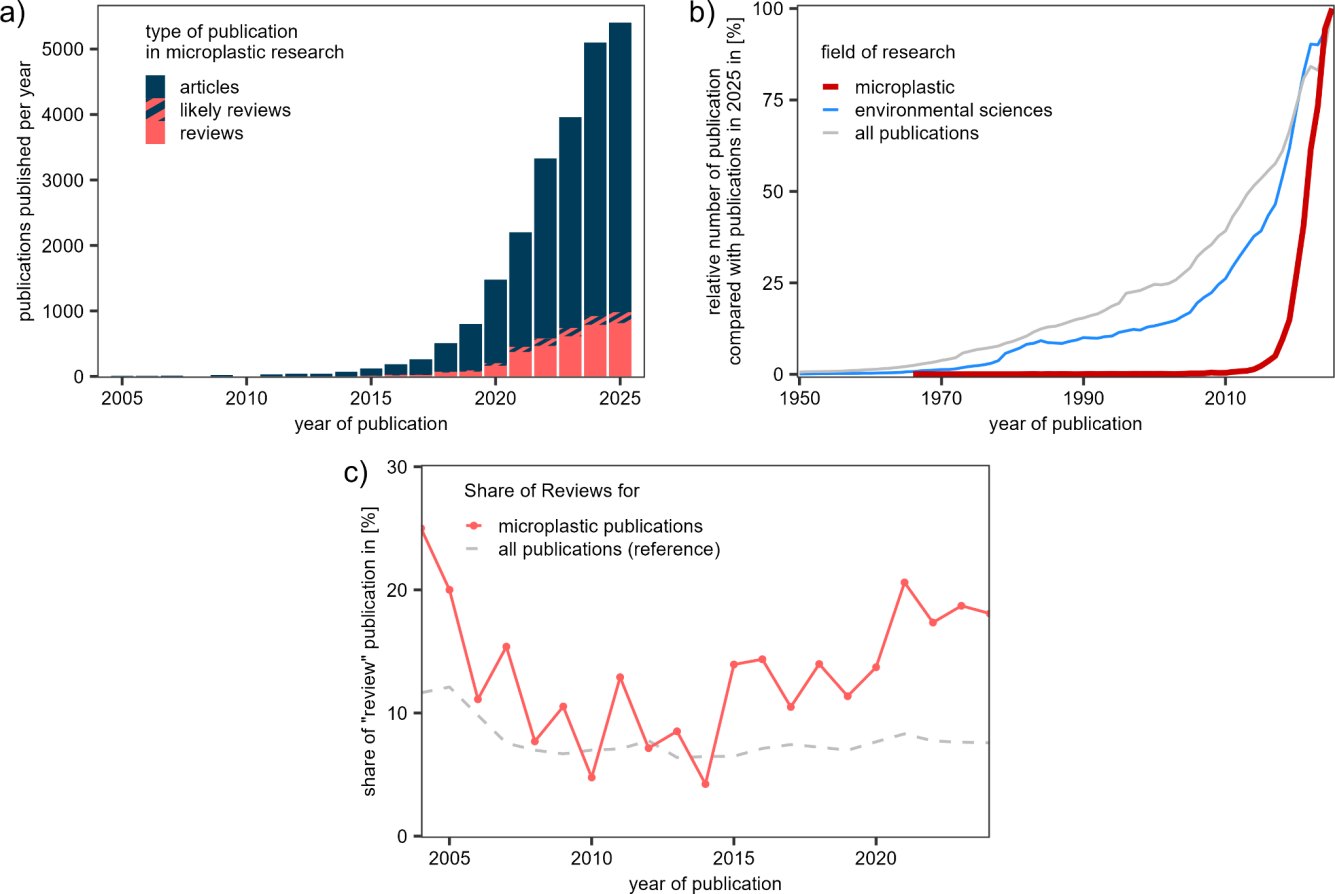
Number of publications on microplastics from 2004–2025 related to the environmental science subject area (a), comparison to other fields (b), the proportion of reviews in microplastics research compared to the overall share of review articles across all scientific publications (c). Publications were retrieved on January 16, 2026 from Scopus using the following search string: ((TITLE-ABS-KEY (microplastic*) OR TITLE-ABS-KEY (micro plastic*)) AND PUBYEAR > 2004 AND PUBYEAR < 2026) AND (LIMIT-TO (DOCTYPE, ″ar″) OR LIMIT-TO (DOCTYPE, ″re″)) AND (LIMIT-TO (LANGUAGE, ″English″)) AND (LIMIT-TO (SUBJAREA, ″ENVI″)). We considered publication as “likely reviews” when the term “review” occurred in the title or the abstract. In b, we normalized the number of publications per research field (classified by Scopus) and all publications. In (c), we compare the share of reviews on microplastics to the share of reviews in the literature in general.

On a practical level, we wonder if and how scientists can keep up with the current publication rate given that the time for reading the literature is limited.^12^ As mentioned, the issue of information overload is neither new nor exclusive to microplastics. In fact, Jean-François Lutz commented in 2012 in Nature Chemistry that “twenty-first-century scientists do not have anything close to the amount of free time that would be necessary to read all of the literature in their field of research, even in very specialized areas”.^16^ Similar voices describe how “scientists are increasingly overwhelmed by the volume of articles bring published”^14^ and offer advice on “how to manage the research-paper deluge”.^12^ On an epistemic level—concerning how knowledge is produced and recognized—we wonder if and how the rapidly expanding literature translates into equally large knowledge gains. In other words, does the scientific community understand microplastics as environmental issue better thanks to the many publications? More importantly, do the resources invested into these publications return knowledge that supports societies in solving the issue at hand?

In this Perspective, we examine this rapid rise in publications more closely, situating it within the broader context of the ‘publish or perish’ culture in academia and other pressures that shape scientific practices. Reflecting on the potential consequences, we argue that the deluge causes information overload that risks generating uncertainty rather than scientific consensus on microplastics. Drawing on Bruno Latour, known for his work on the stabilization of scientific facts,^17^ we highlight the need to move beyond amassing evidence on the problem (e.g., “microplastics are everywhere”) as its existence is already broadly accepted. Yet we argue that numerous extrinsic and intrinsic factors keep the community locked in in its current practices, delaying progress toward a shared understanding, and preventing science from providing orientation knowledge that supports societies in responding to the (micro)­plastics issue.

The aim of this Perspective is to encourage reflection and debate on current publication practices within the microplastics community and its impact on knowledge production. Given that the deluge is a mere symptom of systemic pressures in academia, this includes larger questions on which type of knowledge is most needed to understand and tackle the microplastics issue, and how such evidence is generated and communicated. In our view, these questions are best addressed through collective efforts within the scientific community to find better approaches to identify, prioritize and answer “the big outstanding questions” in microplastics research. With a multitude of pressing ecological crises, combined with politicization of science, deliberate and inclusive reflections on collective direction are both timely and necessary.

## 2 Exploring the Deluge

A simple bibliometric analysis confirms the publication deluge. The literature on microplastics has grown exponentially over two decades (2004–2025), especially over the past ten years (Figure 1a), amounting to more than 23,500 publications in total. This trend in publication activity is not unique to microplastics research: it applies to environmental sciences and to the scientific literature as a whole (Figure 1b). Nevertheless, our analysis indicates that microplastics research represents a particularly pronounced manifestation of this surge. We also find that the proportion of reviews in the field has increased steadily since 2014 (Figure 1c) and has reached almost one-fifth of all publications in the past three years, corresponding to twice as many reviews in microplastics research compared to the overall literature. Our analysis further shows that 2021 (coinciding with the COVID pandemic) saw an increase in the proportion of reviews, reaching a level of up to 20%. In other words, roughly one in five publications on microplastics is currently a review, about twice the proportion seen across the scientific literature overall.

## 3 Surfing the Wave in the Pursuit of Knowledge

The publication deluge raises an important question, namely to what extent it translate into a corresponding increase in our collective understanding of microplastics as an environmental issue and whether this expansion is justified? Since this is a normative and epistemological question, we reflect on the purpose and process of knowledge generation and propose a framework to understand the current status quo that generates this wave of publications (Figure 2). We posit that knowledge generation on microplastics follows three goals, namely, to establish or “stabilize” the problem, to advance a fundamental understanding of the problem, and finally to inform and prioritize solutions.

**Figure 2:**
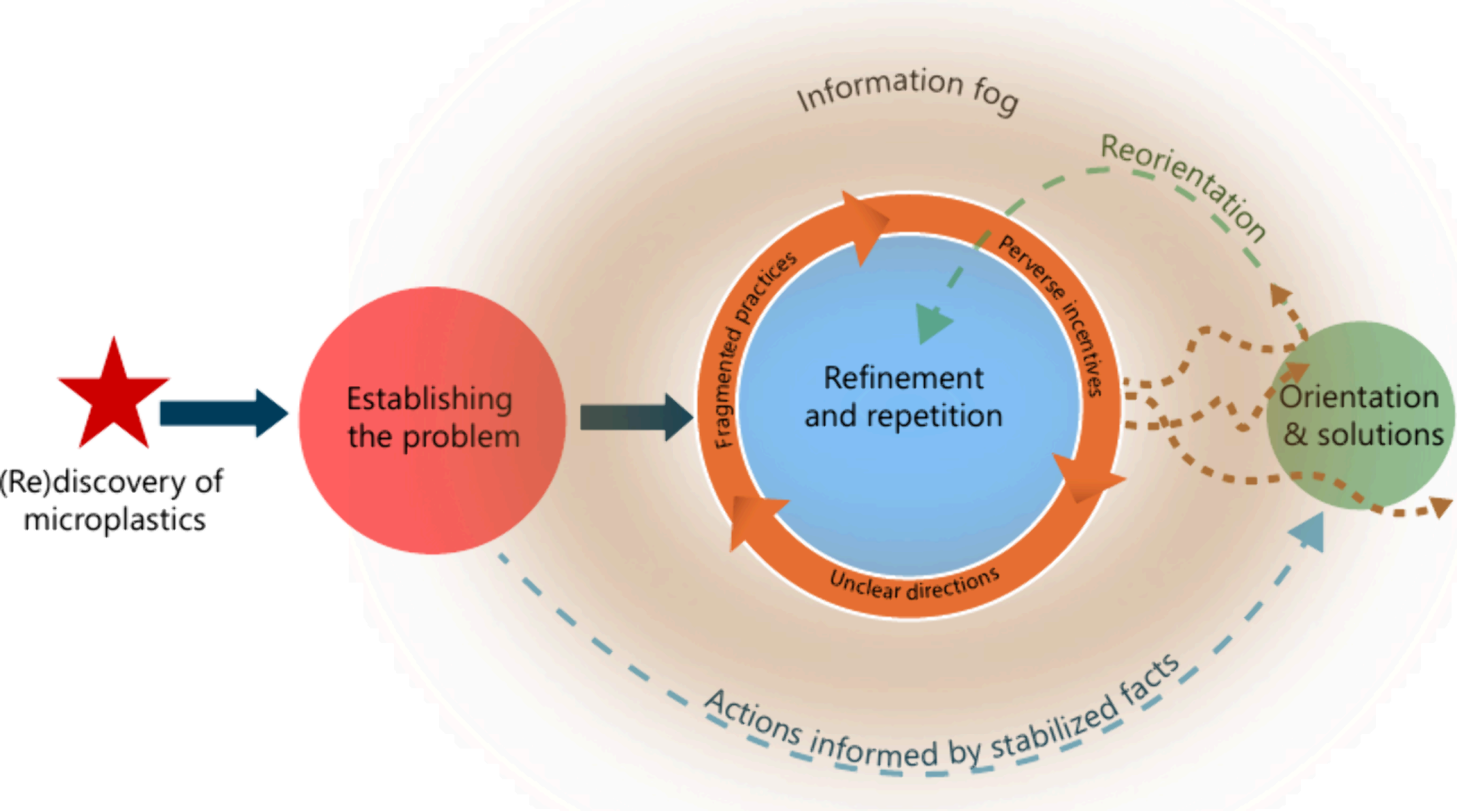
Conceptual framework of knowledge generation in environmental microplastics research. Following the (re)­discovery of microplastics, research initially focused on establishing the existence and scale of the problem (“microplastics are everywhere”). We argue that this has been followed by prolonged refinement and repetition, driven by fragmented practices, perverse incentives (e.g., pressure to publish and hypercompetition for funding, see ref 37 for details), and unclear directions, which generates information fog (as part of the deluge) and impeding convergence toward shared, actionable understanding. Dashed pathways indicate how early findings can trigger immediate orientation and action (blue), how orientation can emerge from the “fog” and lead to more or less suitable solutions (brown), and potential feedback between action and refinement, whereby outcomes reorient inquiry.

### 3.1 Phase 1: Establishing Microplastics as an Environmental Issue

First, and characteristic for the early phase of microplastics research, the main goal was to establish whether microplastics are a problem worthwhile investigating. Driven by the serendipitous discovery of microplastics in the ocean [refs 1–4 and others] such studies typically focused on describing their occurrence in nature but also on their biological uptake and adverse impacts. This has served to establish microplastics as an epistemic object (i.e., an object that can be identified and studied) accessible through operational definitions, analytical and toxicological methods. In a more political sense, this also served to raise concern on an environmental issue that was unknown to society at large.

From our perspective, research succeeded rather rapidly in establishing microplastics as an epistemic object and environmental issue. Despite initial skepticism from peers, arguing that microplastics are a nonissue^18^ or that other environmental problems are more important,^19^ the first phase of research established that microplastics are ubiquitous in nature and have the potential to cause adverse effects in wildlife, both as particles and via associated chemicals. As described in a 2018 book chapter: “Much of what we know can be summarized in three conclusions: fragmented plastic is globally distributed, it is associated with a cocktail of hazardous chemicals and thus is another source of hazardous chemicals to aquatic habitats and animals, and it entangles and is ingested by hundreds of species of wildlife at every level of the food chain including animals we consider seafood”.^20^

While it is difficult to establish when microplastics was broadly accepted as a valid environmental issue, we argue that such an infliction point was reached in the second half of the 2010s, when multiple governments put microplastics legislation into place. Examples include the Microbead-free Waters Act of 2015 in the United States,^21^ the Microbeads regulations of 2017 in the United Kingdom,^22^ and ECHA’s restriction proposal for intentionally added microplastics in the European Union.^23^ Notably, Northrup^24^ identified only one published peer-reviewed study on microbeads in US waterways (i.e., ref 25) available prior to the passage of the Microbead-free Waters Act in 2015. This suggests that action was driven by public concerns rather than a substantial body of evidence. By analogy, microplastics appear to have become an accepted environmental issue roughly a decade ago, when on the order of 900 microplastic papers were available globally. This early work can be seen as having stabilized microplastics as an environmental concern, allowing this understanding to circulate widely in society, and become enshrined in policy and other societal responses to the issue.

### 3.2 Phase 2: Refining Methodologies to Build Trust in the Evidence

In this second phase of research the goal is then to quantify and explore the problem further, fueled by a general curiosity that drives many researchers to understand problems at a deeper level and by policy needs to quantify impacts (e.g., in risk assessments). Epistemically speaking, this phase is *theoretically* oriented toward increasing trust by reducing uncertainty and ambiguity, for instance, via developing more robust and reliable methods, as we have observed in our field. Much emphasis has been put into improving methodologies to quantify microplastics in nature^26^ but the discourse around the environmental realism of toxicity studies also forms part of the broader process of methodological refinement and knowledge consolidation in the field.^6,27^

Efforts to improve methodologies and reduce uncertainty are inherent in the scientific process and, as such, continuous. It is difficult to assess how much progress the community has made in that regard: On the one hand, we have observed the introduction of more sophisticated techniques to quantify microplastics, such as the coupling of microscopic imaging and spectroscopic techniques and mass spectrometry-based methods as well as the emergence of quality criteria for toxicity testing^28–30^ and editorial guidelines.^31^ On the other hand, analyses of the overall quality of microplastics ecotoxicity studies – and their applicability to risk assessment - do not find an improvement over time.^30^ Some subtle improvements to technical quality are observed,^30,32^ but the validity of methods to quantify microplastics remains debated.^33^

The phase of refinement ultimately serves the purpose of increasing scientific and public trust in the evidence on microplastics. As it stands, we argue that such trust has mainly been established by the sheer amount of research—that is the deluge—rather than through methodological advancement, scientific consolidation and knowledge synthesis. Certainly, the community has made important methodological progress. Yet much of that effort has focused on describing the problem better in analytical terms, while advances in understanding have been comparatively limited, or obscured by the deluge. Epistemologically speaking, method refinement has become decoupled from questions of understanding and precision has become a substitute for insight.

### 3.3 Phase 3: From Knowledge to (In)­action

One can argue that microplastics research, similar to other areas of environmental sciences, operates in postnormal conditions, in such that scientific facts are deeply intertwined with values and ecological urgency.^34^ Accordingly, it follows a mode of “crisis epistemology” where microplastics are entangled with nature, technology, economy and politics and characterized by high uncertainty and potentially high stakes. Under such conditions, the philosopher Bruno Latour argues that providing orientation in an uncertain and transforming world becomes more central than classical modes of science aimed at understanding phenomena in great detail.^17^

We argue that research has indeed generated significant orientation knowledge on microplastics by stabilized key facts, such as their ubiquity, persistence and impacts. Even though uncertainties and blind spots remain, this knowledge renders inaction increasingly untenable and has already prompted some societal responses. However, caught in a cycle of repetition, the literature amounts to the deluge that, in turn, generates information fog (Figure 2). Such fog is characterized not only by weak or repetitive studies but also robust or novel work that is not being synthesized into coherent orientation knowledge.

Rather than building trust and providing orientation, we argue that this fog creates confusion that threatens the knowledge gains the community has made and places scientific uncertainties at the heart of communication. This creates a perceived lack of converging findings that exposes the field to undue criticism. It can also create confusion about, and competition between, solutions (Figure 2). Ultimately this results in a haphazard approach to solution, resulting in the promotion of (bio)­degradability, for instance, without sufficient reflection on whether this will actually reduce or exacerbate the microplastics problem.

Ultimately, the surge in scientific publications can therefore do the exact opposite of what is intended: fostering distrust and inflating uncertainty beyond what the evidence indeed warrants. This risks eroding scientific authority on the matter, rendering established knowledge contestable again, and supports the agenda of actors with vested economic interests that stand to gain from a state of confusion that obstructs societal action.^35^ Accordingly, the repercussions of the deluge reach beyond academia, with potential implications for policymaking and other responses to microplastics.

## 4 Propelling the Deluge

We believe that the deluge cannot be attributed to bad-faith science. While emphasis is often placed on moral failures on behalf of individual scientists, we contend that the underlying causes are systemic and rooted in a much deeper epistemic crisis.

Contemporary societies are characterized by a constant flow of information with media outlets, advertising and social media constantly competing for our attention. The political landscape has shifted toward simplified narratives, in which populist messages offer “quick fixes” to complex problems. In other words, attention has become a commodity, for which different actors compete. The same applies to research,^36^ where noticeability becomes not only a prerequisite for funding and career progression but also for one’s work to be read. We stipulate that, as a result, highly publicized studies, often offering simplified narratives, will disproportionately shape public perceptions of the field, overshadowing more nuanced evidence.

Perverse incentives in academia^37^ are thus the driving forces propelling the deluge, promoting a “publish or perish”, metrics-driven culture that rewards rapid and excessive publishing at the expense of knowledge synthesis. This system is further perpetuated by a corporate publishing industry that profits from quantity rather than quality and hence enables the rise of paper mills and generative artificial intelligence creating more epistemic fog. Besides upholding the pressure to publish, and thereby further fueling the deluge, funding agencies will tend to support conventional, low-risk research.^38,39^ Thereby, they will be more inclined to support repetitive and descriptive work on microplastics, promoting a “more of the same”-type of research, rather than guiding research toward the important questions and supporting knowledge synthesis.

## 5 Rebuilding after the Deluge

### 5.1 Raise the Bar

Alongside earlier arguments by Provencher et al.,^15^ there is a clear need to increase study quality, ensure that new publications are relevant for the broader field, and promote contextualized, hypothesis-driven studies as drivers in microplastics research. In addition, we see a strong need for better knowledge synthesis in purpose-driven systematic reviews and meta-analysis that cut through the fog and address larger and relevant research questions.

This approach can counter the publication deluge by raising the bar for the production and acceptance of scientific outputs. This can be achieved by agreeing on quality criteria for microplastics studies as a community. However, this strategy can also inadvertently reinforce exclusivity in research if “quality” can only be achieved through access to advanced instrumentation, highly specialized expertise or resource-intensive methodologies. This comes with a high risk of marginalizing researchers and institutions with limited resources and may contribute to forms of academic colonialism, where knowledge production becomes increasingly concentrated in rich regions and laboratories. Addressing the publication deluge therefore requires not only higher standards, but also inclusive definitions of quality that go beyond technical sophistication. For example, value should be placed also on synthesis, contextual relevance, and solid study design.

### 5.2 Promote Alternative Forms of Data Sharing

We acknowledge that calls for fewer scientific publications may be perceived as discouraging scientific productivity. However, we argue that this need not be the case; rather, it reflects a reprioritization of scientific effort. For example, while stand-alone monitoring studies may be categorized as instances of “refinement and repetition” (Figure 2), this does not imply that such studies are without value. The data outputs from such studies can, for instance, create value through meta-analyses, contribute to modeling or machine learning approaches, or support local action on microplastics. We question, however, that such work needs to be published in the peer-reviewed literature and suggest other formats would be more fit-for-purpose, in particular deposition of results in open repositories with strong reporting and quality requirements. In other cases, relevant end-users of the specific data are best reached through context-specific gray literature reports.

Several efforts have been made to establish shared data repositories for data on microplastics occurrence in the environment^40–43^ and others are currently underway.^44^ We fully support these initiatives and advocate for broad adoption. However, to see them gain traction, the community will need to build consensus around shared standards for data curation and actively promoted their use and acceptance. Importantly, such data sharing platforms should expand beyond specific domains (e.g., NOAA database for marine environments,^41^) to encompass diverse environmental compartments and scientific field. Also, data sharing practices need to be further strengthened to ensure that more research data is findable, accessible, interoperable, and reusable (FAIR), as highlighted in a recent bibliographic analysis^45^. Promoting and incentivizing such alternative routes of data publication would reduce the fog in microplastics literature.

### 5.3 Change the Publishing Culture

The historical development of the scientific publishing industry has been described elsewhere (e.g., ref 46), detailing the rise and fall of scientific journals. Beale^46^ further alludes to the role of scientific journals as gatekeepers in a competitive academic landscape, where publication metrics were introduced as proxies for quality and prestige. These dynamics are compounded by the contemporary attention economy, as described above, which places additional pressure on publication output. The culture of *“publish or perish”* is now widely recognized. Together, these structures create incentives for opportunistic publishing practices, including *salami slicing* of data into *smallest publishable unit*, thereby further fueling the publication deluge.

Addressing the issue requires systemic changes in the scientific community at large, shifting norms on the purpose and mode of publishing. The arrival of generative artificial intelligence may mark the beginning of the end of the current publication system, forcing a fundamental shift in how scientific knowledge is produced and evaluated.^46^ Amidst these transformative changes, we argue that all actors in the publishing system, foremost the scientific community, can take actions to create a healthier publishing culture.

Authors can decide to shift from publishing smallest publishable units of descriptive and repetitive research toward richer studies that are hypothesis-driven and synthesize larger chunks of data, so they provide the orientation knowledge needed. This transition should be led by established researchers because early career researchers (ECRs) are more vulnerable to the “publish or perish” culture and often face publication-based requirements for Ph.D. completion and career advancement (e.g., h-index thresholds). It is therefore imperative that senior authors, including ECR supervisors, actively support and protect such shifts in research priorities.

This change must be accompanied by institutional support, for example through reduced publication pressure (e.g., fewer publication requirements for Ph.D. degrees), evaluation systems that reward quality over quantity (e.g., diversified assessment criteria in hiring and promotion), and explicit recognition of gray literature and data deposition as valuable scientific outputs. Not all scientifically useful work needs to be published as a peer-reviewed article.

Editors and reviewers can more actively promote a shift in publishing culture. They can improve on their gatekeeping role by providing clear guidance on which type of work is sought for, and which not. They can reject overly descriptive or redundant studies, while providing transparent and constructive reasoning for these decisions. This could go hand in hand with establishing clear quality and reporting guidelines. They can resist the pressure from publishers to increase publication rates and decrease publishing times. Making such change in practices can support a collective move toward a healthier publishing system.

Publishers also have a role to play in reducing overall publication volume. As many current business models are driven by publication throughput, powerful incentives prevent changes in these practices. However, the scientific community could take back control by establishing more society-owned, nonprofit journals, and publish in those. Notably, some high-end publishers already demonstrate that alternative publishing models are feasible, where quality outweighs quantity. An additional measure would be to place less emphasis on rapid time-to-decision, allowing for more thorough, constructive and open peer review.

Finally, funding bodies hold the true power to change. They shape the research landscape and can guide the field toward less descriptive and more synthetic research directions. This includes targeted support for hypothesis-driven research on microplastics, as well as dedicated funding for data synthesis, integration, and theory building. Importantly, funders should support the scientific community in establishing new directions and practices, so change becomes a systemic, rather than top-down.

### 5.4 An Argument for Slow Science

At this point we invite readers to reflect on their own scientific practice: when was the last time you read scientific papers in depth without a predefined objective? When was the last time you discussed fundamental scientific questions with a colleague without an expectation of a tangible outcome? When was the last time you felt genuinely scientifically creative? If these moments are difficult to recall, this, in our opinion, reflects a clear structural problem in how scientific work is currently organized, rather than a failure of individual motivation or effort.

Over time, a corporate model of scientific productivity has become normalized, reinforced by funding structures that often prioritize short-term deliverables. This leaves researchers feeling more like providers of predefined solutions than independent thinkers with time and space to explore fundamental questions. At the same time, academia has become a high-stress profession, with mounting concerns over researcher well-being and mental health.^47,48^ There are, however, encouraging signs of a shift that challenge this trajectory, including the Slow Science movement^49–51^ and initiatives such as the Coalition for Advancing Research Assessment,^52^ which seek to realign scientific practice with reflection, quality, and meaningful, iterative and sustainable knowledge generation, recognizing a diverse range of outputs and contributions in support of impact. As stated in the Slow Science Manifesto: scientists “do need time to think”, “time to digest” and “time to misunderstand each other”, especially when fostering dialogue.^50^ This also implies valuing understanding over output, quality over quantity, collaboration over competition.

Promoting institutional adoption of CoARA principles and Slow Science approaches would not only support a healthier academic environment but could also help counter the publication deluge and associated epistemic fog, thereby enhancing the societal relevance of scientific research. This applies both to microplastics research and to science more broadly.

This Perspective does not intend to pass judgment from an ivory tower but rather expresses our wish to improve the scientific ecosystem which we are part of. As a research community, it remains important to support openness and plurality in microplastics research. At the same time, the scientific community needs to find new approaches to determine shared directions and practices. The *format* of how we generate and communicate knowledge on microplastics may need to evolve, but the need for both remains essential. We are conscious of the irony inherent in contributing yet another publication to the body of literature and of our own role in literature inflation as authors, reviewers, supervisors, and editors. Acknowledging this shared responsibility, we offer these ideas not as prescriptions, but as a starting point for reflection and—hopefully—change.

## ⊥

Egli Engineering AG, 9000 St. Gallen, Switzerland
